# Circadian Regulation Patterns With Distinct Immune Landscapes in Gliomas Aid in the Development of a Risk Model to Predict Prognosis and Therapeutic Response

**DOI:** 10.3389/fimmu.2021.797450

**Published:** 2022-01-06

**Authors:** Ruotong Tian, Yimin Li, Minfeng Shu

**Affiliations:** ^1^ Department of Pharmacology, School of Basic Medical Sciences, Shanghai Medical College, Fudan University, Shanghai, China; ^2^ Department of Pathology, Fudan University Shanghai Cancer Center, Shanghai, China; ^3^ Department of Oncology, Shanghai Medical College, Fudan University, Shanghai, China; ^4^ Ministry of Education (MOE) & Ministry of Health (MOH) Key Laboratory of Medical Molecular Virology, School of Basic Medical Sciences, Shanghai Medical College, Fudan University, Shanghai, China

**Keywords:** glioma, circadian rhythm, tumor microenvironment, prognostic model, therapy

## Abstract

Circadian disruption in tumorigenesis has been extensively studied, but how circadian rhythm (CR) affects the formation of tumor microenvironment (TME) and the crosstalk between TME and cancer cells is largely unknown, especially in gliomas. Herein, we retrospectively analyzed transcriptome data and clinical parameters of glioma patients from public databases to explore circadian rhythm-controlled tumor heterogeneity and characteristics of TME in gliomas. Firstly, we pioneered the construction of a CR gene set collated from five datasets and review literatures. Unsupervised clustering was used to identify two CR clusters with different CR patterns on the basis of the expression of CR genes. Remarkably, the CR cluster-B was characterized by enriched myeloid cells and activated immune-related pathways. Next, we applied principal component analysis to construct a CRscore to quantify CR patterns of individual tumors, and the function of the CRscore in prognostic prediction was further verified by univariate and multivariate regression analyses in combination with a nomogram. The CRscore could not only be an independent factor to predict prognosis of glioma patients but also guide patients to choose suitable treatment strategies: immunotherapy or chemotherapy. A glioma patient with a high CRscore might respond to immune checkpoint blockade, whereas one with a low CRscore could benefit from chemotherapy. In this study, we revealed that circadian rhythms modulated tumor heterogeneity, TME diversity, and complexity in gliomas. Evaluating the CRscore of an individual tumor would contribute to gaining a greater understanding of the tumor immune status of each patient, enhancing the accuracy of prognostic prediction, and suggesting more effective treatment options.

## Introduction

Gliomas represent 80% of all primary brain tumors and are a heterogeneous group of lethal tumors of the central nervous system (CNS) (1). In 2016, the newly established World Health Organization (WHO) classification of CNS tumors added molecular genetic features including isocitrate dehydrogenase (IDH) and chromosomal 1p/19q status to the previous version, which solely histologically classified gliomas into four grades (2). Despite very comprehensive treatment protocols consisting of debulking surgery, chemotherapy, and concomitant radiotherapy (3), the median survival of grade IV glioblastoma multiforme (GBM) is only 12–14 months (4). A remarkable level of genetic, epigenetic, and environmental heterogeneity existing within each individual glioma explains multiple mechanisms of therapeutic resistance and forms a highly resilient disease (5). A comprehensive model based on gliomas integrated with TME will be of benefit to predicting prognosis and selecting drugs and meet the demand of individualized treatment.

Cancer immunotherapies advancing from the trial stage to established first- or second-line indications have revolutionized the treatment of solid tumors. However, clinical trials of glioma immunotherapy are not proceeding well. The limited response of glioma to immunotherapy may be dependent on the tumor-intrinsic characteristics and the brain microenvironment. The specific TME of gliomas, characterized by the low frequency of T cells, the high frequency of myeloid cells, and the lower expression of cell-surface inhibitory markers, makes it challenging for immune checkpoint blockade (ICB) to exert a strong therapeutic effect (6–8). Meanwhile, a biomarker to optimize patient selection is most urgently needed due to a lack of broad ICB approval.

Circadian rhythm is a conserved phenomenon that governs a large array of physiological and metabolic functions. The mammalian circadian rhythm depends on a time-delayed transcription translation feedback loop (TTFL). The aryl hydrocarbon receptor nuclear translocator-like protein 1 (ARNTL; also named brain and muscle ARNT-like protein 1, BMAL1) and circadian locomotor output cycles kaput (CLOCK) constitute the positive arm (9, 10), which can promote the expression of cryptochrome (CRY1 and CRY2) and period (PER1, PER2, and PER3). As the negative arm of the TTFL, CRYs and PERs form a complex to suppress the BMAL1–CLOCK complex (11, 12). As for the second feedback loop, the BMAL1–CLOCK complex regulates the expression of nuclear receptors REV-ERBα/β (also known as nuclear receptor subfamily 1, group D, members 1/2, NR1D1/2) and retinoic acid receptor-related orphan receptors (RORs), which in turn repress and activate BMAL1, respectively (13, 14). Over the past several decades the connection between circadian clocks and tumorigenesis has been well studied. Abnormal rhythms are associated with high-grade brain tumors (15, 16). Specifically, disruption of the circadian clock pharmacologically (REV-ERB agonists) or genetically (CLOCK and BMAL1 short hairpin RNAs) impairs glioma stem cell (GSC) stemness in GBM and causes GSC cell-cycle arrest and apoptosis (17, 18). As for TME, high CLOCK levels in GSCs correlate with a high frequency of microglia *via* the regulation of olfactomedin-like 3 and correlate with a decreased level of activated CD8+ T cells (18). Therapeutically, in view of cyclical expression patterns of DNA repair genes (19), circadian rhythms influence therapeutic sensitivity and thus play prominent roles in regulating the antitumor efficiency of chemotherapy (20). These findings suggest targeting clock-regulated crosstalk between TME and cancer cells will help to develop a novel prognosis signature and effective clock-oriented immunotherapies that may have synergistic effects with conventional therapies to increase the effectiveness of glioma treatment.

This bioinformatics analysis is intended to explore circadian rhythm-controlled tumor heterogeneity and characteristics of TME in gliomas. The dataset of glioma in The Cancer Genome Atlas (TCGA) was used as the training set and that in the Chinese Glioma Genome Atlas (CGGA) was used as the validation set throughout the study. Unsupervised clustering identified two stable CR patterns with different characteristics of infiltrated immune cells, activated signaling pathways and immune response processes. PCA algorithm was used to construct the CRscore that could not only be an independent factor to predict the prognosis of glioma patients but also guide them to choose suitable treatment strategies: immunotherapy or chemotherapy. In this study, we revealed that glioma immune landscapes regulated by two distinct circadian patterns renewed our cognition to improving individualized therapy based on clock within gliomas.

## Methods

### Data Acquisition and Preprocessing

The RNA sequencing data of TCGA-LGG (Lower Grade Glioma; WHO grade II–III), TCGA-GBM (glioblastoma multiforme; WHO grade IV), and GTEx-brain in transcripts per kilobase million (TPM) format uniformly processed by TOIL were downloaded from UCSC xena (https://xenabrowser.net/datapage/). The corresponding clinicopathological data were obtained from the cBioPortal website (http://www.cbioportal.org/). Patients without survival information were excluded in a further study. The genomic mutation data (including somatic mutation and copy number variation) of TCGA-LGG and TCGA-GBM were downloaded from the Genomic Data Commons (https://portal.gdc.cancer.gov/) using the “TCGAbiolinks” R package (21). Somatic mutation data were analyzed using “maftools” R package, and significant amplifications or deletions of copy number were detected using GISTIC 2.0. To obtain the CGGA validation set, the RNA sequencing data and related clinicopathological data were downloaded from the CGGA website (https://www.cgga.org.cn). Two independent datasets, including one with 325 individuals and another with 693 individuals, were acquired. Batch effects from non-biological technical biases were corrected using the “ComBat” algorithm of the “sva” R package (22).

### Unsupervised Clustering for Circadian Rhythm Genes

Six CR gene sets were concluded from the Molecular Signatures Database (MSigDB) (23), KEGG pathways (24), REACTOME database (25), GO biological processes (GO: 0007623 CIRCADIAN RHYTHM), WikiPathways (26), and review literatures (27–29). The gene annotated in not less than two CR gene sets was defined as a CR gene for further integrated analysis and modeling. A total of 91 acknowledged CR genes were curated and analyzed to identify distinct CR patterns (**Table S1** and **Figure S1**). The CR gene widely described in reviews is defined as a core CR gene. Unsupervised clustering analysis was applied to identify CR patterns based on the expression of 91 CR genes from TCGA or CGGA and classify patients for further analysis. A consensus clustering algorithm was performed using the “ConsensuClusterPlus” R package and was repeated 1,000 times for guaranteeing the stability of classification (30).

### Gene Set Variation Analysis and Gene Ontology Annotation

To investigate the difference in biological process between CR patterns, we performed GSVA enrichment analysis using the “GSVA” R package (31). The gene sets of “h.all.v7.4.symbols” and “c2.cp.kegg.v7.4.symbols” were downloaded from the MSigDB database to run GSVA analysis (http://www.gsea-msigdb.org/gsea/downloads.jsp). The GSVA scores among different circadian patterns were determined using the “limma” R package. The threshold was set as the adjusted *p*-value < 0.05 and the absolute (log_2_ Fold change, FC) > 0.1. The “clusterProfiler” R package was used to perform functional annotation for CR-related genes, and the top 20 GO annotations were shown (32).

### Estimation of TME Cell Infiltration and Signatures

As we did in our previous study, the “ESTIMATE” algorithm was used to calculate Tumor Purity, ESTIMATE Score, Immune Score, and Stromal Score (33, 34). Furthermore, we used the ssGSEA (Single-sample Gene Set Enrichment Analysis) algorithm, CIBERSORT algorithm (35), and xCELL algorithm (36) to quantify the relative abundance of each type of cells infiltrating the glioma TME. The gene sets of “TME-associated signatures” were downloaded and analyzed using the “IOBR” R package (37). The ssGSEA method was chosen in the process of signature score evaluation. TIP (Tracking Tumor Immunophenotype) is a meta-server that systematically integrates two existing third-party methods “ssGSEA” and “CIBERSORT” for tracking, analyzing, and visualizing the status of anti-cancer immunity and the proportion of tumor-infiltrating immune cells across a seven-step cancer-immunity cycle using RNA-seq or microarray data (38). The correlations between the CRscore and the steps of the cancer-immunity cycle were analyzed using the “ggcor” R package.

### Generation of the CRscore

The construction of CRscore was performed as follows: firstly, the differentially expressed genes (DEGs) associated with the CR cluster phenotype were determined using the “limma” R package. Specifically, gene expression data were normalized by voom and then fed to lmFit and eBayes functions to calculate the differentially expressed statistics. The significance filtering criteria of DEGs were set as an adjusted *p*-value < 0.05 and |log_2_(FC)| ≥1.5. Then, the prognostic analysis was performed for each DEGs using a univariate Cox regression model. A total of 719 genes with significant prognosis were extracted for further analysis using the “randomForestSRC” R package. We also used the “randomSurvivalForest” algorithm to rank the importance of 719 genes, and the gene with a relative importance >0.2 was selected to construct the CR signature (39). We then curated the expression profile of the final 50 determined genes to perform PCA, and principal components 1 and 2 were extracted and served as the signature score. We then adopted a formula similar to previous studies (40), CRscore = Σ(*PC*1_*i*_ + *PC*2_*i*_), where *i* is the expression of 50 prognostic-related CR cluster DEGs.

### Immuno-/Chemotherapeutic Response Prediction

The Tumor Immune Dysfunction and Exclusion (TIDE) algorithm (41) and Immune Cell Abundance Identifier (ImmuCellAI) algorithm (42) were used to predict response to ICB therapy. We also used immunotherapeutic cohorts with complete clinical information to predict patients’ response to ICB therapy. In the GSE78220 cohort, the data of patients with metastatic melanoma treated with pembrolizumab, an anti-PD-1 antibody, were downloaded from Gene Expression Omnibus (GEO), and the FPKM data of gene expression profiles were converted to the more comparable TPM value among samples. We also used the “Prophetic” R package to predict the chemotherapy response of each sample based on Genomics of Drug Sensitivity in Cancer (GDSC) (https://www.cancerrxgene.org/). The correlation between estimated IC_50_ and CRscore was established using Spearman correlation analysis with thresholds as |Spearman *R*| > 0.5 and *p*-value < 0.05.

### Statistical Analyses

For comparison between two groups, statistical significance for normally distributed variables was estimated by Student’s *t*-tests, and nonnormally distributed variables were analyzed by the Wilcoxon rank-sum test. For comparison among three or more groups, one-way ANOVA and Kruskal–Wallis test were used for normally or nonnormally distributed variables, respectively. Fisher’s exact test was performed for categorical data. Correlation coefficients were computed by Spearman and distance correlation analyses. The “surv-cutpoint” function from the “survival” R package was applied to stratify samples into CR-high and -low groups. Kaplan–Meier curves and the Log-Rank test were adopted to assess whether there were differences in overall survival among groups. Univariate and multivariate Cox regression analyses were utilized to evaluate the independent prognostic value of the CRscore regarding OS, which were revealed by forest maps. We next performed multivariate Cox regression to establish a nomogram; the survival predictive accuracy of prognostic models was assessed based on the calibration plots. All statistical *p*-values were two-sided, with *p* < 0.05 considered as statistically significant. All statistical tests were performed in R statistical software (v4.05, R Core Team, R Foundation for Statistical Computing, Vienna, Austria).

## Results

### Landscape of Genomic Variations and Transcription Profile of Circadian Rhythm Genes in Glioma

**Figure 1:**
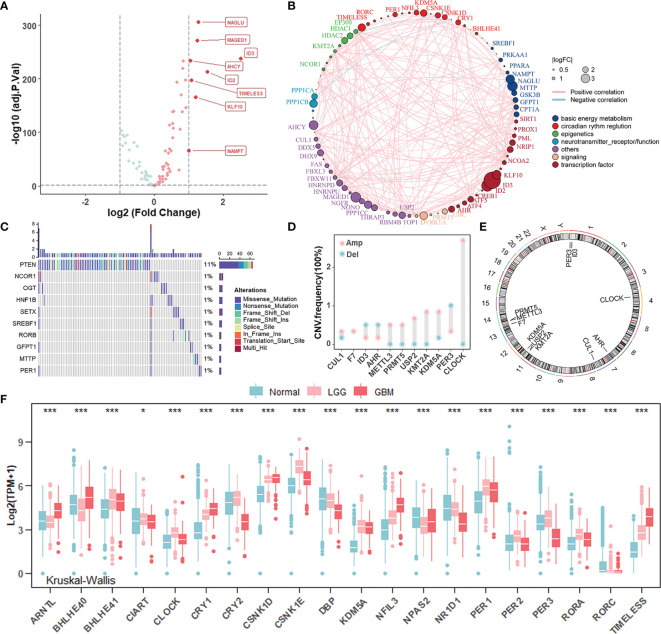
Landscape of genomic variations and transcription profile of circadian rhythm genes in gliomas. **(A)** Volcano plot of differentially expressed CR genes between normal and glioma (TCGA-LGGGBM: TCGA-LGG and TCGA-GBM) samples (Wilcoxon test: adjust *p* < 0.05, and |log2FC| > 1). The overexpressed genes in gliomas were highlighted in red. **(B)** The interaction between CR genes in gliomas. The CR genes with different features were depicted by circles in different colors. The lines connecting CR genes represented their interaction with each other. The size of each circle represented the differential expression of CR genes in gliomas compared to normal. **(C)** The mutation frequency of CR genes in 584 patients from the TCGA cohort. Each column represented an individual patient. The upper bar plot indicated mutation-accumulation. The bar plot on the right indicated the proportion of each variant type with the number above representing mutation frequency. Only top 10 mutations were included. **(D)** The CNV frequency of CR genes in glioma patients from the TCGA cohort. The height of each column represented the alteration frequency. The amplification frequency, pink dot; The deletion frequency, blue dot. **(E)** The location of CR genes with CNV alteration on chromosomes. **(F)** The mRNA expression level of core CR genes among normal, TCGA-LGG, and TCGA-GBM samples. Normal, blue; LGG, pink; GBM, red. The asterisks represented the statistical *p*-value (Kruskal–Wallis test: *p < 0.05; ***p < 0.001).

To investigate whether there were specific expression patterns of CR genes in gliomas, we determined several differentially expressed CR genes between glioma and normal samples using the “limma” R package (**Figure 1A**). The genes involved in negative transcription regulation (ID3, ID2, and KLF10), cell metabolism (NACLU and AHCY), and cell survival (MAGED1 and TIMELESS) were upregulated in tumors when |log_2_FC| ≥ 1 and adj.*p* ≥ 0.05 were set as thresholds. Furthermore, the comprehensive landscape of interaction among CR genes and feature groups to which they belong was depicted in the **Figure 1B** network, and we noticed that most of the CR genes were positively correlated with each other. To investigate whether genomic variation contributed to abnormal CR gene expression in gliomas, we described mutations and copy number variations (CNVs) of CR genes, respectively. In the top 10 mutations of CR genes, only PTEN exerted 11% mutant frequency, suggesting that genetic mutations of CR genes might not be the main cause of rhythm perturbations (**Figure 1C**). The vast majority were mutations in PETN, a common tumor suppressor gene, which could be included here because of its expression governed by circadian rhythms. Besides, the CNV frequency showed that CLOCK, the positive arm of TTFL, gained copy number, while PER3, a part of negative arm of TTFL, had a frequency of CNV deletion (**Figure 1D**). Thus, we have come to know that CNV partly conduced to disturbed rhythms of tumors. The location of CR genes with CNVs on chromosomes is shown in **Figure 1E**. To explore whether the core regulators of circadian TTFLs had unique expression patterns in gliomas, we analyzed the mRNA levels of core CR genes among normal, LGG, and GBM samples (**Figure 1F**). It was worth noting that major factors (CRYs and PERs) of negative arms were upregulated in LGG compared to normal, but with relatively low expression in GBM. Given all this, the disruption of circadian rhythms due to the high heterogeneity of genomic variations and transcription profile covertly contributed to tumorigenesis and progression of gliomas.

### Identification of Circadian Patterns Mediated by CR Genes

**Figure 2:**
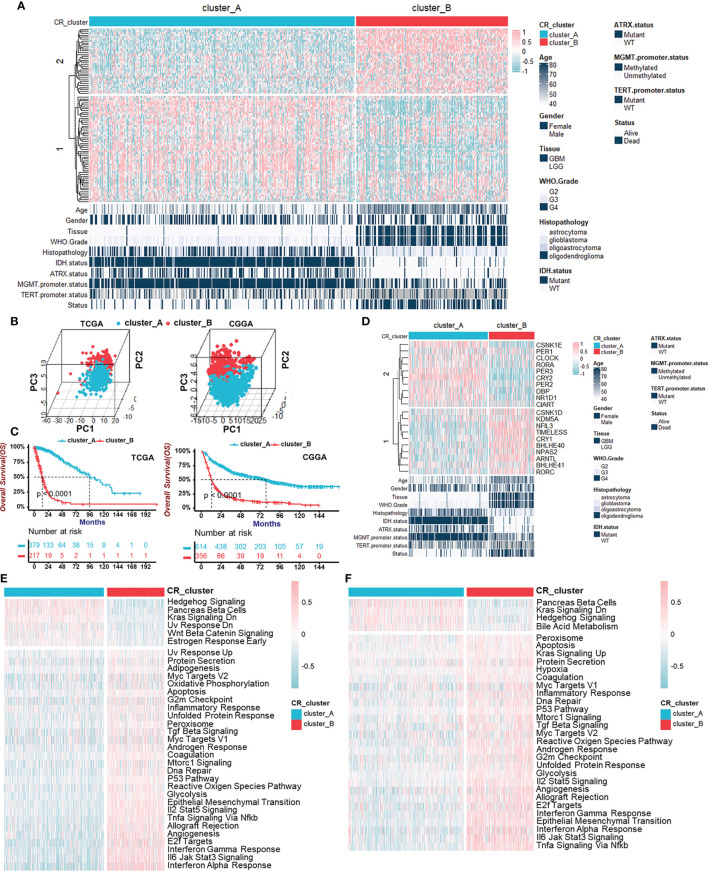
Identification of two circadian patterns mediated by CR genes in TCGA cohort. **(A)** Unsupervised clustering of 91 CR genes for 596 glioma patients in the TCGA cohort resulted in two CR clusters. Age, gender, tissue, WHO grade, histopathology, IDH status, ATRX status, MGMT promoter status, TERT promoter status, and survival status are shown as patient annotations. Pink represented the relatively high expression of CR genes and blue represented the relatively low expression. **(B)** Principal component analyses for the transcriptome profiles of CR patterns in TCGA (left) and CGGA (right) cohorts, respectively, showing a remarkable difference on transcriptome between two CR patterns. **(C)** Survival analyses for CR patterns in the TCGA cohort (left) including 379 cases in CR cluster-A and 217 cases in CR cluster-B (Log-Rank test: *p* < 0.0001), and in the CGGA cohort (right) including 614 cases in CR cluster-A and 356 cases in CR cluster-B using Kaplan–Meier curves (Log-Rank test: *p* < 0.0001). **(D)** The transcriptome of core CR genes with remarkable differences in two CR patterns corresponded with the previous clustering. Pink represented the relatively high expression of core CR genes and blue represented the relatively low expression. **(E, F)** GSVA enrichment analyses in two CR clusters showing the activation states of Hallmark pathways (MSigDB) in TCGA **(E)** and CGGA **(F)** cohorts. Activated pathways, pink; Inhibited pathways, blue.

Given the aberrant expression of certain circadian regulators in gliomas, we wondered whether there were distinctively different CR patterns, on the whole, among individual tumors. The unsupervised hierarchical cluster analysis was used to uncover circadian patterns, and the TCGA cohort population was separated into two distant clusters, termed CR cluster-A and -B, by the “ConsensusClusterPlus” R package (**Figures 2A**, **S2A–C**). Additionally, similar results obtained in the CGGA cohort verified our findings above (**Figures S3A–C**). Moreover, principal component analysis confirmed that CR genes could distinguish two CR clusters perfectly, in both TCGA and CGGA cohorts (**Figure 2B**). Obvious discrepancy between two CR clusters was embodied in histopathology features and patients’ prognoses. Patients in CR cluster-A were characterized by lower grades and harmless molecular genetic features, which coincided with particularly prominent survival advantage (**Figures 2C**, **S2D, E**). The same results were obtained in LGG and GBM groups, respectively (**Figures S2D**, **S3D**). We also added the unsupervised clustering results in LGG and GBM, respectively. Unsupervised clustering could also divide LGG patients into two groups, and there was a significant difference in prognosis between the two groups (**Figures S2F–I**). As for core CR genes, the distribution of their transcriptome levels with remarkable differences between two clusters corresponded with the previous clustering (**Figure 2D**). ARNTL (BMAL1) and CLOCK together constitute the positive arm of TTFLs, but they took advantages of expression level in different patterns. Similarly, the components of the negative arm were expressed preferentially.

Next, to explore the global functions in two CR patterns beyond the individual and single gene expression, we performed GSVA enrichment analyses, which showed the activation states of Hallmark pathways (MSigDB) in TCGA (**Figure 2E**) and CGGA (**Figure 2F**) cohorts. Hedgehog Signaling activation, Pancreas beta cells, and Kras Signaling Downregulation were observed in CR cluster-A, while immune response-related pathways consisting of “Interferon Response”, “Interleukin Signaling”, and “Tnfα Signaling *via* Nfκb” and pathways associated with malignant biological behaviors including “Epithelial-Mesenchymal Transition” and “Angiogenesis” were activated in CR cluster-B. KEGG pathway enrichment analyses in TCGA (**Figure S3F**) and CGGA (**Figure S3G**) cohorts also supported the activated immune reaction-related pathways. From above, we identified two internally gathered circadian patterns and functional enrichment analyses recognized CR cluster-B closely related to the immune response.

### Characterization of the Immune Cell Infiltration in Distinct Circadian Patterns

**Figure 3:**
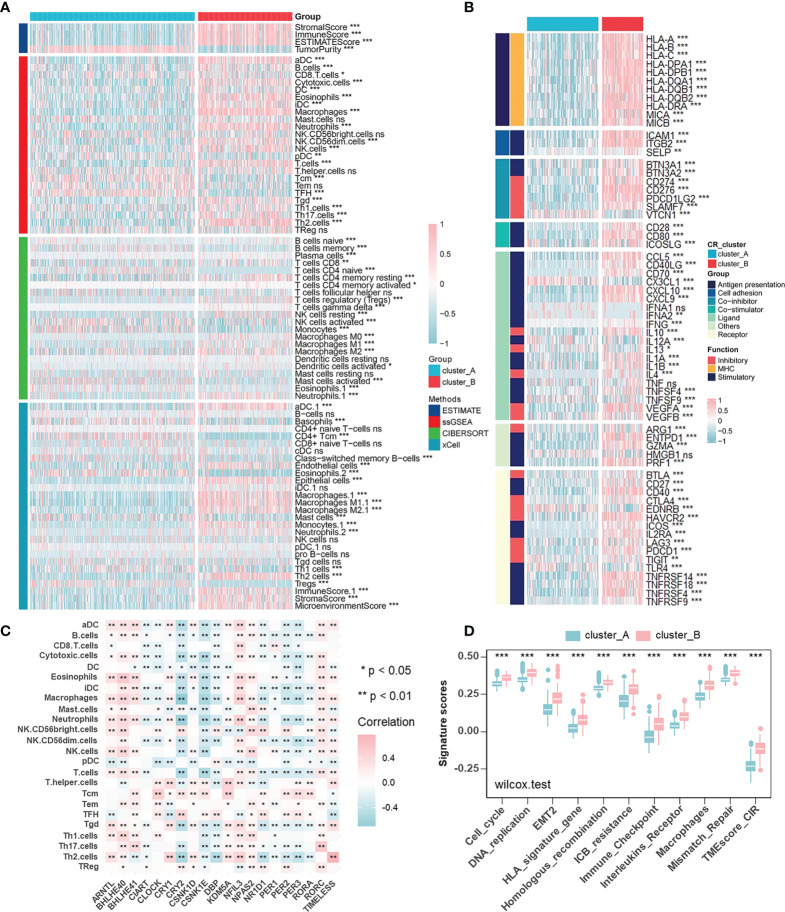
Characterization of the immune cell infiltration in distinct circadian patterns. **(A)** Heatmap of TME cell infiltration characteristics in two CR patterns assessed by four different methods. **(B)** Heatmap showing the differences of immune-related gene expression in two CR patterns. Upregulation, pink; Downregulation, blue. **(C)** The correlation between TME infiltration cell type and each core CR genes using spearman analyses. Negative correlation, blue; Positive correlation, pink. **(D)** CR clusters were distinguished by different TME relevant signatures and TME score (Wilcoxon test: ***p < 0.001).

To describe immune status in different CR patterns, we utilized four different methods (ESTIMATE, ssGSEA, CIBERSORT, and xCELL) for evaluating immune cell infiltration (**Figure 3A**). ESTIMATE algorithm showed that CR cluster-B exhibited high Stromal and Immune Scores and low Tumor Purity, which represented a significantly increased immune cell infiltration and generally indicated a relatively hot tumor immune microenvironment. However, in gliomas, components of immune system enriched in CR cluster-B were macrophages (ssGSEA, CIBERSORT, and xCELL), dendritic cells (ssGSEA and xCELL), neutrophils (ssGSEA, CIBERSORT, and xCELL), endothelial cells (xCELL), and Th2 cells (ssGSEA and xCELL), generally considered as immunosuppressive components in tumors. Additionally, CR cluster-B was mainly characterized by relatively high expressions of human leukocyte antigens (HLA) and immune checkpoint molecules (**Figure 3B**). On the one hand, class I HLA, expressed on the surface of almost all nucleated cells, could increase the possibility of presenting more immunogenic antigens and of benefiting from ICB. High immune checkpoint expression also implied effective anti-immune checkpoint therapy. On the other hand, in line with the characteristics of immune cell infiltration, class II HLA molecules, mainly expressed on the surface of antigen-presenting cells (dendritic cells, B cells and macrophages), and many inhibitory molecules (IL-4, IL-10, and VEGF) were unregulated in CR cluster-B. We then examined the specific correlation between each TME infiltration cell type and each core CR gene using Spearman’s correlation analyses (**Figure 3C**). The correlation focused on the relationship between immunosuppressive cells (macrophages, neutrophils, and T helper cells) and the core TTFL components (ARNTL, BHLHE40/41, CRY2, and PER2/3). Furthermore, CR clusters could be distinguished by different TME relevant signatures and TME score (**Figure 3D**). The above results revealed that immunosuppressive components enriched in CR cluster-B suggested an immunosuppressive microenvironment and coincided with poor prognoses of patients.

### Generation of Circadian Gene Clusters and Functional Annotation

**Figure 4:**
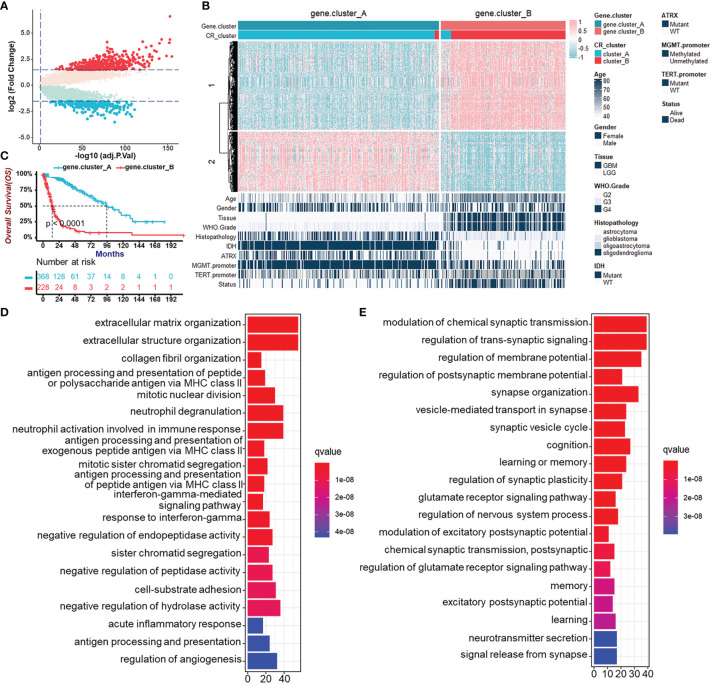
Generation of circadian gene clusters and functional annotations for CR-related genes. **(A)** Volcano plot of CR pattern-related DEGs between CR cluster-A and -B (Wilcoxon test: adjust *p* < 0.05, and |log2FC| > 1.5). **(B)** Unsupervised clustering of CR pattern-related DEGs to classify patients of the TCGA cohort into different subtypes, termed CR gene cluster-A and -B, respectively. The gene clusters, CR clusters, and other parameters were used as patient annotations. **(C)** Survival analysis for CR gene clusters in the TCGA cohort including 368 cases in gene cluster-A and 228 cases in gene cluster-B using Kaplan–Meier curves (Log-Rank test, *p* < 0.0001). **(D)** Functional annotation for CR-related genes upregulated in gene cluster-B (Gene Set 1 in **B**) using GO enrichment analysis. The length of the bar plots represents the number of genes in that category. The color depth represented *q*-value. **(E)** Functional annotation for CR-related genes upregulated in gene cluster-A (Gene Set 2 in **B**) using GO enrichment analysis. The length of the bar plots represents the number of genes in that category. The color depth represented *q*-value.

To further validate CR regulation patterns and investigate the potential biological behavior of CR patterns, we determined 719 genes, which were DEGs between CR cluster-A and -B and also related to prognosis of glioma patients using the “limma” R package and univariate Cox regression analysis (**Figure 4A**). Then, we performed unsupervised clustering analyses based on the obtained CR-related DEGs. Consistent with the clustering grouping of previous CR patterns, the unsupervised clustering algorithm also revealed two distinct CR-related signatures termed CR gene cluster-A and -B, respectively (**Figure 4B**, **Figures S4A, B**). This confirmed that two distinct CR patterns did exist in gliomas. Survival analysis for CR gene clusters in the TCGA cohort using Kaplan–Meier curves suggested survival advantages in CR gene cluster-A no matter in all glioma samples (**Figure 4C**) or in TCGA-LGG and TCGA-GBM, separately (**Figure S4C**). Next, the “clusterProfiler” R package was used to perform GO enrichment analysis for Gene Set 1 that were upregulated in gene cluster-B (**Figure 4D**), and Gene Set 2 upregulated in gene cluster-A (**Figure 4E**). GO’s annotations for Gene Set 1 fell into several categories including antigen presentation, inflammation, and immune responses, while the annotations for Gene Set 2 focused on neurotransmitter transmission and other major duties of CR genes controlling the brain. These analyses confirmed that gene cluster-B was significantly associated with immune-relevant signatures, whereas gene cluster-A was associated with regulations of synaptic transmission. Besides, the expression of core CR genes (**Figure S4D**), the infiltration of immune cells (**Figure S4E**), and Hallmark (**Figure S4F**) as well as KEGG (**Figure S4G**) pathways in two CR gene clusters were consistent with the previous clustering results. The results of secondary clustering verified that there were indeed two CR-based patterns in gliomas.

### Construction of the CRscore and Exploration of Its Immunological Relevance

**Figure 5:**
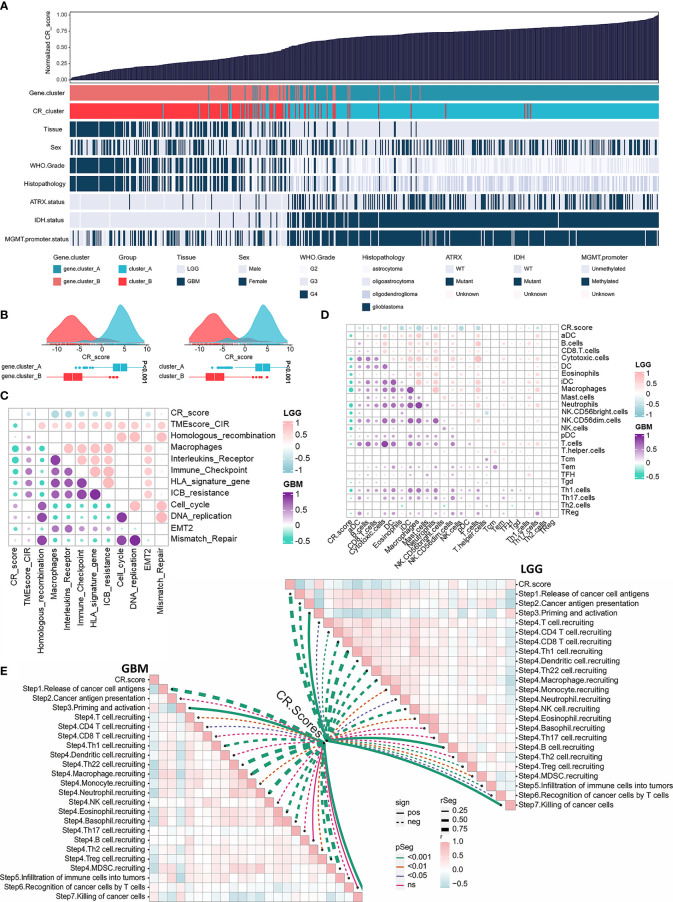
Construction of the CRscore and exploration of its immunological relevance. **(A)** An overview of the association between CRscores and other patient annotations in TCGA cohort. **(B)** Comparison of CRscores between gene cluster A and B in the TCGA cohort, *p* < 0.001 (left); Comparison of CRscores between CR cluster A and B in the TCGA cohort, *p* < 0.001 (right). **(C)** The correlations between CRscore and TME relevant signatures in LGG as well as GBM of TCGA cohort, respectively (*p* > 0.05). Pink represented positive correlation and blue represented negative correlation in LGG; purple represented positive correlation and green represented negative correlation in GBM. **(D)** The correlations between CRscore and ssGSEA scores of TME cells in LGG as well as GBM of the TCGA cohort, respectively (*p* > 0.05). Pink represented positive correlation and blue represented negative correlation in LGG; purple represented positive correlation and green represented negative correlation in GBM. **(E)** The correlations between CRscore and steps of the cancer immunity cycle in LGG (right) as well as GBM (left) of TCGA cohort, respectively.

Considering the intratumoral heterogeneity and complexity of circadian disruption, based on 50 prognostic-related CR cluster DEGs, we developed a score termed CRscore using the PCA algorithm. The general distribution of CRscore, CR clusters, gene clusters, and molecular pathological parameters in the TCGA cohort were described in the heatmap (**Figure 5A**). Low CRscore crowded in CR cluster-B and gene cluster-B (**Figure 5B**). Considering that the immune microenvironment of LGG and GBM might differ, we explored the immunological relevance of CRscore separately in LGG and GBM. The correlation was supported by TME-related pathways and immune cell infiltration analyses and even more pronounced in LGG (**Figures 5C, D**). Next, we explored the indicating effect of CRscore in the anti-tumor immune process (**Figure 5E**) and discovered that CRscore was likely to be associated with the recruitment of immune cells. Some steps including priming and activation of immune cells; recruiting Th1, Th22 cells, and macrophages; and tumor killing effect were positively correlated with CRscore in both LGG and GBM. Peculiarly, in steps significantly correlated with the CRscore, a low score in GBM is related to recruitment of immunosuppressive cells (neutrophils, eosinophils, basophils, Tregs, and MDSCs). A low score in LGG indicated enhanced recruitment of anti-tumor immune cells (CD8+ cells, Th1 cells, and DCs).

### Evaluation of the Prognostic Potentiality of the CRscore

**Figure 6:**
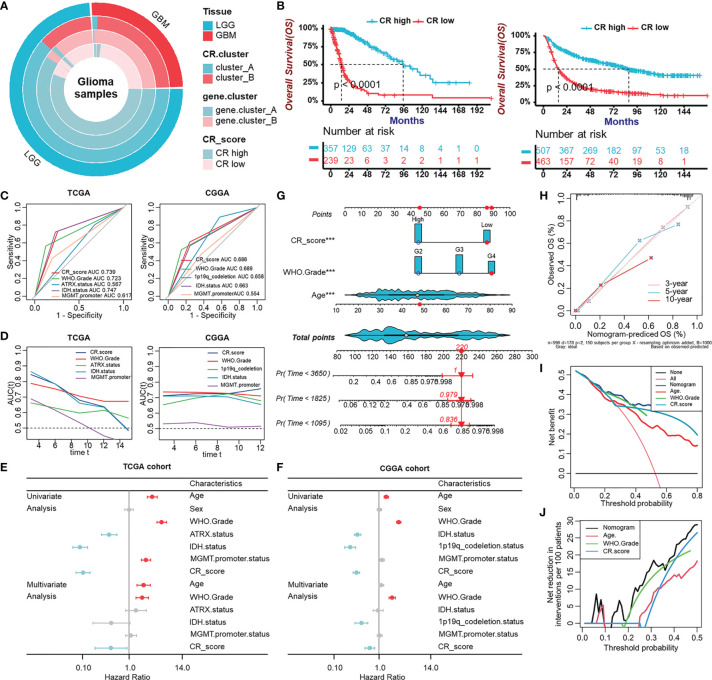
Evaluation of the prognostic potentiality of the CRscore. **(A)** The overlap among tissue types, CR clusters, gene clusters, and CR groups. **(B)** Survival analyses for CR groups in the TCGA cohort (left) including 357 cases in the CR-high group and 239 cases in the CR-low group (Log-Rank test, *p* < 0.0001), and in the CGGA (right) cohort including 507 cases in the CR-high group and 463 cases in the CR-low group using Kaplan–Meier curves (Log-Rank test, *p* < 0.0001). **(C)** Predictive accuracy at 5-year of CRscore compared with other histological or molecular indicators in TCGA (left) and CGGA (right) cohort, respectively. The accuracy was equal to the area under the ROC curves (AUC). **(D)** Time-dependent AUC of CRscore compared with other histological or molecular indicators in the TCGA (left) and CGGA (right) cohort, respectively. **(E, F)** Univariate and multivariate analyses of clinicopathological characteristics and CRscore with overall survival in the TCGA **(E)** and CGGA **(F)** cohort, respectively. **(G)** Nomograms for predicting the probability of patient mortality based on WHO Grade, CRscore, and age. **(H)** Plots depicted the calibration of nomograms. **(I)** Net decision curve analyses demonstrating the benefit for predicting overall survival on nomogram. **(J)** The net reduction analyses demonstrated in how many patients the nomogram could be avoided without prognosis of miscalculation.

After having identified the CRscore as an intrinsic pattern closely linked to the immune process, we sought to determine whether the CRscore could accurately predict outcomes of glioma patients. Firstly, the overlap among tissue types, CR clusters, gene clusters, and CR groups was described in **Figure 6A**. The 596 patients in the TCGA cohort were assigned to two groups (CR-high and -low) based on CRscores using the cutoff value (0.4) obtained with the “survminer” R package. Survival analysis indicated that the CR-high group had prolonged survival time in the TCGA cohort as well as in LGG or GBM (**Figure S5A**), which was further validated in the CGGA cohort (**Figures 6B**, **S5B**). The area under the ROC curves (AUC) at 5 years (**Figure 6C**) and time-dependent AUC (**Figure 6D**) of the CRscore compared with other histological or molecular indicators in the TCGA and CGGA cohort, respectively, verified the predictive efficiency of the CRscore. When the CRscore was evaluated as a categorical variable (high or low CRscore) in the Cox regression model, the CRscore was determined to be an independent and robust prognostic factor. Univariate and multivariate analyses of clinicopathological characteristics and CRscore in the TCGA and CGGA cohort are shown in **Figures 6E, F**, respectively. Furthermore, we established a prognostic nomogram to predict 3-, 5-, and 10-year overall survival based on the stepwise Cox regression model. WHO grade, CRscore, and age were included in the prediction model (**Figure 6G**). The C-index of the nomogram was 0.864 (95% CI, 0.840–0.888). Nomogram prediction and actual observation in the TCGA cohort reached an excellent agreement at the 3-, 5-, and 10-year survival probability after calibration (**Figure 6H**). Net decision curve and the net reduction analyses demonstrated the superiority of this nomogram in predicting prognosis (**Figures 6I, J**).

### Landscape of Tumor Somatic Mutation in Two CR Groups

**Figure 7:**
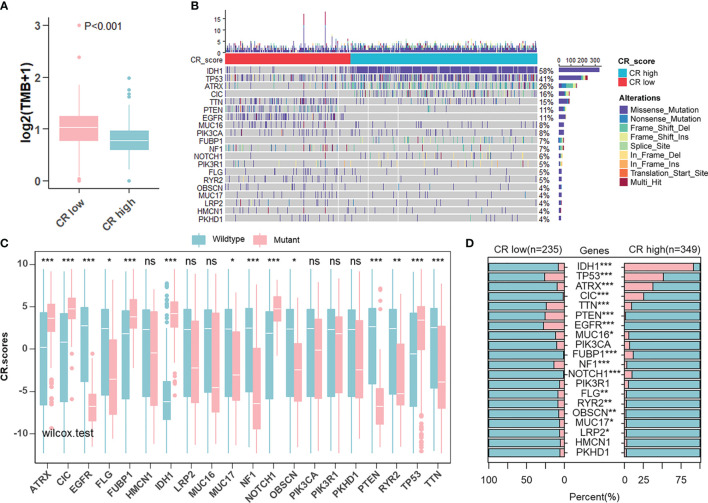
Landscape of tumor somatic mutation in two CR groups. **(A)** Differences of TMB in CR-high and -low groups in TCGA cohort (Wilcoxon test: *p* < 0.001). **(B)** Top 20 most frequent mutations in patients from TCGA cohort and the distribution of mutations in CR high and low groups. **(C)** Differences in CR scores between wild-type and mutant groups of each of the top 20 genes in the TCGA cohort (Wilcoxon test: ns, P>0.05; *p < 0.05; **p < 0.01; ***p < 0.001). **(D)** Differences in the frequency of top 20 mutations between CR-high and -low groups in the TCGA cohort (Fisher’ exact test: *p* > 0.05; *p < 0.05; **p < 0.01; ***p < 0.001).

Given that the aim of exploring the value of the CR pattern on treatment and the response to ICB are closely related to somatic mutation, we decided to analyze differences in somatic mutation between CR-high and -low groups in the TCGA cohort using the “maftools” R package. As shown in **Figure 7A**, the CR-low group presented more extensive tumor mutation burden (TMB), on the whole, than the high-score group. Differences in CRscores between wild-type and mutant groups of each gene inversely proved the negative correlation between mutation and score (**Figures 7B, C**). The mutation occurrence varied between two groups. In detail, genetic mutations of IDH1, TP53, ATRX, and CIC mainly appeared in the CR-high group, while TTN, PTEN, and EGFR appeared in the CR-low group (**Figure 7D**).

### The Role of the CRscore in the Prediction of Therapeutic Benefits

**Figure 8:**
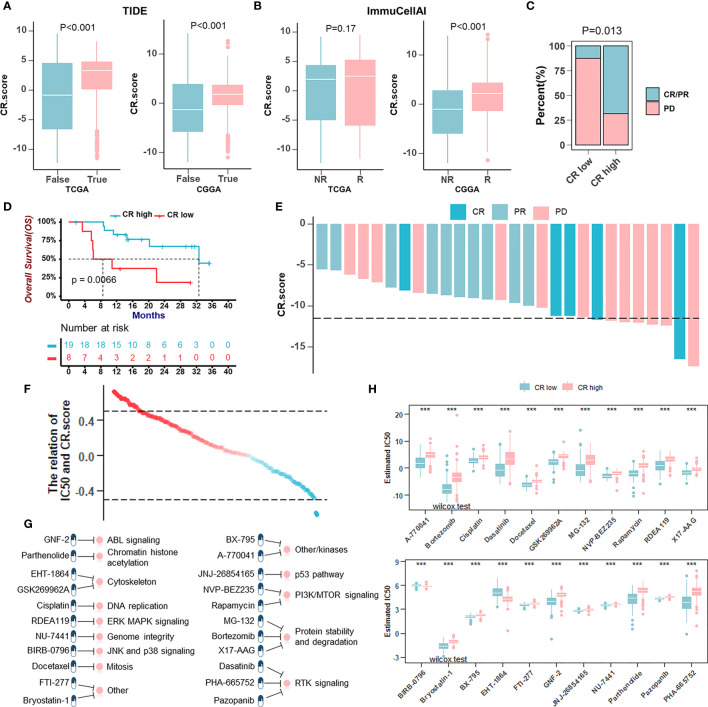
The role of the CRscore in the prediction of therapeutic benefits. **(A, B)** The relative distribution of TIDE **(A)** in TCGA (*p* > 0.001) and CGGA (*p* > 0.001) cohorts and ImmuCellAI **(B)** in TCGA (*p* = 0.17) and CGGA (*p* > 0.001) cohorts were compared between CR score high versus low groups, respectively. **(C)** The proportion of patients’ response to PD-1 blockade immunotherapy in CR-high or -low groups. PD, progressive disease; CR, complete response; PR, partial response (Fisher’ exact test: *p* = 0.013). **(D)** Survival analyses for low (8 cases) and high (19 cases) CR score patient groups in the anti-PD-1 immunotherapy cohort (GSE78220) using Kaplan–Meier curves (Log-Rank test: *p* = 0.0066). **(E)** The correlation of CRscore with clinical response to anti-PD-1 immunotherapy. **(F)** The correlation between CRscore and the estimated IC_50_ for drugs evaluated by the Spearman analysis. Each point represents a drug. **(G)** Pathways targeted by candidate drugs. **(H)** The box plots of the estimated IC_50_ for candidate drugs in CR-low and -high groups (Wilcoxon test: ***p < 0.001).

Newly identified predictors, such as TIDE (**Figure 8A**) and ImmuCellA (**Figure 8B**), are widely used to evaluate the immune response. Our analysis revealed that a lower CRscore was not only associated with a poorer response to ICB but also more prone to immune escape. In anti-PD-1 immunotherapy cohort (GSE78220), a survival benefit trend was observed in patients with high CRscore (**Figures 8C–E**). To further understand the effects of the CRscore on drug response, we assessed the association between the CRscore and the response to drugs in cancer cell lines. Using the Spearman correlation analysis, we identified twenty-two drugs targeting various pathways (**Figure 8G**) significantly correlated between CRscores in the Genomics of Drug Sensitivity in Cancer (GDSC) database (**Figure 8F**). Among them, twenty agents with lower IC_50_ in the CR-low group might be beneficial to low CRscore patients, while two agents might be beneficial to patients with high scores (**Figure 8H**). Together, these results implied that CR pattern played a crucial role in mediating the immune response and was also correlated with drug sensitivity. Thus, the CRscore might be a potential biomarker for establishing appropriate treatment strategies.

## Discussion

Circadian disruption is associated with tumorigenesis and tumor progression through effects on cancer cell biological properties, including proliferation, DNA repair, apoptosis, metabolism, and stemness. Emerging evidence indicates that circadian clock also plays an influential role in the TME. Abnormalities of clock components (e.g., CLOCK, ARNTL/BMAL1, PER, CRY, RORs, and REV-ERBα) in tumors could reshape TME by regulating the expression and secretion of soluble factors including HIF1α, ARNT, VEGF, OLFML3, and other unidentified factors (27). These factors modulated TME biology, including endothelial cell biology such as promoting angiogenesis and antiangiogenic therapy resistance, the infiltration of myeloid cells, as well as the infiltration and activation/suppression of lymphocytes. Clock components in immune cells, in turn, affected tumor growth. BMAL1 in macrophages inhibited the production of ROS and HIF1α, and affected tumor growth through regulating macrophage alternative polarization (43) RORα and RORγ in T cells (Th17 cells and CD8+ T cells) could modulate their differentiation and activation, which affected tumor growth and the antitumor immune response (44–46). The above studies pointed out the importance and necessity of studying the clock-regulated crosstalk between TME and cancer cells.

On the basis of the CR gene list concluded from six gene sets, we performed the first unsupervised clustering in glioma patients from TCGA and CGGA cohorts, respectively, and identified two CR clusters with different composition and expression of core CR genes, which were considered as different CR patterns. The CR cluster-B showed a unique immune status characterized by high infiltration of immune cells, high expression of immune-related genes, and highly activated immune-related pathways, which suggested that circadian rhythms might affect the biological behavior of tumor itself, as well as the infiltration and function of immune cells, thus affecting the prognosis. Considering that the pro-tumoral pathway was activated and the immunosuppressive cells were infiltrated, it was reasonable that the outcomes of the patient were relatively poorer in CR cluster-B. CSNK1E, shown to phosphorylate PERs, and PERs were all downregulated in the CR cluster-B group, which were all negatively correlated with the infiltration of most immune cells. BHLHE40/41 repressing CLOCK-ARNTL’s transactivation of PER1 corresponds to the opposite expression of BHLHE40/41 and PER1 at mRNA levels. In view of the correlation between RORγ (RORC) and Th17/T cells has been confirmed by experimental studies (45, 46), correlations among genes or between a single gene and a certain type of immune cell might uncover the mechanism of circadian rhythms regulating tumor immune microenvironment, which needed to be explored further.

To construct a CR-based prognostic signature, a second unsupervised clustering was performed based on the DEGs between CR cluster-A and -B. Two distinct CR gene clusters (CR gene cluster-A and -B) proved that two stable CR patterns did exist in gliomas. Next, we constructed CRscore by using PCA algorithm and established the relationship between CRscore and immune signatures. Analyses of immune components and anti-tumor processes provided the reason why prognoses of patients in low score group were poor. CRscore was negatively associated with recruitment and infiltration of immune cells, as well as the pathways related to TME. However, there were some subtle and critical differences between LGG and GBM. In LGG, a lower CRscore was related to enhanced recruitment of immune cells but diminished killing tumor effect, characterized by immune disability. In GBM, a lower CRscore represented recruitment of immunosuppressive cells, characterized by immune suppression. Therefore, in both LGG and GBM, CRscore was negatively correlated with prognosis. More accurate prognostic evaluation method was established by univariate and multivariate regression analysis as well as nomogram and validated.

As neo-antigens result from mutations, the more neo-antigens that are present, the higher the TMB. With more neo-antigens present, there may be an increased probability that some of the neo-antigens presented by MHC proteins will be immunogenic (47, 48). This is the root of the hypothesis that cancers with high TMB are more likely to benefit from ICBs. Yet, approximately 60% of patients with high TMB do not respond to ICBs (49). Similarly, in this study, the CR-low group with more extensive TMB did not gain advantages in ICB treatment, and even the opposite result was obtained. Our results confirmed the theory that TMB had limitations as a predictive biomarker, especially when used in isolation (50). As mentioned above, patients with lower CRscores not only tended not to respond to ICBs but also were more prone to immune escape when using TIDE and ImmuCellA to evaluate the immune response. In other cancer cohorts treated with ICB (GSE78220), the CR-low group showed a higher progression disease ratio. However, in drug sensitivity analysis, the CR-low group was sensitive to more drugs, suggesting another treatment strategy. Recent advances of the glioma TME highlight the complex and immunosuppressive environment within the tumor that underlies the resistance to immunotherapy. According to the characteristics of glioma, ICB strategies different from other solid tumors need to be developed urgently. Macrophages found within the glioma tend to be immunosuppressive and are associated with poor survival, suggesting that tumor-associated macrophage or microglia (TAM)-targeting therapies (alone or in combination with other therapies) altering TAM characteristics or abundance may improve immunotherapeutic outcomes (6, 51). Strategies to enhance therapy response to ICB might thus involve the mechanism-driven combination of ICB and targeting of TAMs.

Although we reviewed the databases and literatures to curate a catalog of 91 recognized CR genes, a series of newly identified or side CR genes need to be incorporated into the model to optimize the accuracy of the CRscore. Besides, the CR patterns and the CRscore were identified by using retrospective datasets; thus, the role of CR score in immunotherapy needs to be confirmed by more indicators, and more prospective cohorts of glioma patients receiving immunotherapy are needed to validate our findings.

In conclusion, we comprehensively evaluated and verified the CR patterns among 1,566 glioma samples based on recognized CR genes, and systematically correlated these patterns with TME characteristics. This integrated analysis indicated that circadian rhythm upset laid a critical foundation for understanding crosstalk between TME and tumor cells in gliomas. More broadly, evaluating the CRscore of the individual tumor will contribute to enhancing our cognition of the characteristics of TME and provide important insight into therapeutic efficacy.

## Data Availability Statement

Publicly available datasets were analyzed in this study. These data can be found here: TCGA (The Cancer Genome Atlas); CGGA ( Chinese Glioma Genome Atlas).

## Author Contributions

MS, RT, and YL contributed to the study conception. RT was responsible for writing the first draft of the paper. RT and YL conducted the data analysis. MS was responsible for funding acquisition and supervision. All authors contributed to the article and approved the submitted version.

## Funding

This work was supported by the National Natural Science Foundation of China (Project 81872467 and 82073880), the Shanghai Municipal Health Commission (Program 201940233), and the Program for Professor of Special Appointment (Eastern Scholar) at Shanghai Institutions of Higher Learning (Program TP2019047).

## Conflict of Interest

The authors declare that the research was conducted in the absence of any commercial or financial relationships that could be construed as a potential conflict of interest.

## Publisher’s Note

All claims expressed in this article are solely those of the authors and do not necessarily represent those of their affiliated organizations, or those of the publisher, the editors and the reviewers. Any product that may be evaluated in this article, or claim that may be made by its manufacturer, is not guaranteed or endorsed by the publisher.

## Supplementary Material

Supplementary Figure 1 Six CR gene sets were concluded from five datasets and review literatures. The gene annotated in not less than two circadian gene sets was defined as a CR gene. A total of 91 acknowledged CR genes were concluded here. The number of pick circles connected by lines indicated the number of times a CR gene appears in different gene sets; The number of genes sharing the same annotation pattern was marked at the top of the corresponding bar chart. The bar chart on the left showed the number of circadian genes in each gene set.

Supplementary Figure 2 Identification of two circadian patterns mediated by CR genes in TCGA cohort. **(A)** CDF plot showing a real random variable of its probability distribution based on consensus scores for each k (from 2 to 6, indicated by different colors) in TCGA cohort. **(B)** Consensus matrices of TCGA cohort for each k (k = 2–6), displaying the clustering stability using 1000 iterations of hierarchical clustering. **(C)** Survival analyses for CR patterns in TCGA cohort using Kaplan–Meier curves for OS when k = 3–6 (Log-Rank test: P < 0.0001). **(D)** Kaplan–Meier curves for OS of TCGA-LGG cohort (upper) including 372 cases in CR cluster-A and 72 cases in CR cluster-B (Log-Rank test: P < 0.0001), and TCGA-GBM (lower) including 7 cases in CR cluster-A and 145 cases in CR cluster-B (Log-Rank test: P = 0.0085). **(E)** The proportion of representative clinical parameters of patients in CR cluster A and B relevant to **Figure 2A** (Fisher’ exact test: P <0.001). **(F)** Consensus matrices of TCGA-LGG cohort for k = 2, displaying the clustering stability using 1000 iterations of hierarchical clustering. **(G)** Kaplan–Meier curves for OS of patients in TCGA-LGG cohort when k = 2. **(H)** Consensus matrices of TCGA-GBM cohort for k = 2, displaying the clustering stability using 1000 iterations of hierarchical clustering. **(I)** Kaplan–Meier curves for OS of patients in TCGA-GBM cohort when k = 2.

Supplementary Figure 3 Verification of two circadian patterns in CGGA cohort and functional annotations of CR clusters. **(A)** CDF plot showing a real random variable of its probability distribution based on consensus scores for each k (from 2 to 6, indicated by different colors) in CGGA cohort. **(B)** Consensus matrices of CGGA cohort for each k (k = 2–6). **(C)** Kaplan–Meier curves for OS of CGGA cohort when k = 3–6 (Log-Rank test: P < 0.0001). **(D)** Kaplan–Meier curves for OS of LGG patients in CGGA cohort (upper) including 476 cases in CR cluster-A and 120 cases in CR cluster-B (Log-Rank test: P < 0.0001), and GBM patients in CGGA cohort (lower) including 138 cases in CR cluster-A and 236 cases in CR cluster-B (Log-Rank test: P < 0.0001). **(E)** Unsupervised clustering of 91 CR genes for 970 glioma patients in CGGA cohort resulted in two CR clusters. Age, gender, tissue, WHO grade, IDH status, 1p/19q codeletion status, MGMT promoter status, and survival status are shown as patient annotations. **(F, G)** GSVA enrichment analyses in TCGA **(F)** and CGGA **(G)** cohorts showing the activation states of KEGG pathways in two CR clusters. Activated pathways, pink; Inhibited pathways, blue.

Supplementary Figure 4 Generation of circadian gene clusters and functional annotations of CR gene clusters. **(A)** Consensus matrices of TCGA cohort for k = 2. **(B)** CDF plot showing a real random variable of its probability distribution based on consensus scores for each k (from 2 to 6, indicated by different colors) in TCGA cohort. **(C)** Kaplan–Meier curves for OS of TCGA-LGG patients (left) and TCGA-GBM patients (right), respectively. **(D)** The mRNA expression level of core CR genes in gene cluster A and B. Gene cluster A, blue; Gene cluster B, pink. The asterisks represented the statistical p value (Wilcoxon test: ns, P >0.05; *, P <0.05; ***, P <0.001). **(E)** The ssGSEA score of TME cells in two CR gene clusters of TCGA cohort. (Wilcoxon test: ns, P>0.05; **, P <0.01; ***, P <0.001). **(F, G)** GSVA enrichment analyses in TCGA cohort showing the activation states of Hallmark pathways (MSigDB) **(F)** and KEGG pathways **(G)** in two CR gene clusters. Activated pathways, pink; Inhibited pathways, blue.

Supplementary Figure 5 Survival analyses for CR groups. **(A)** Survival analyses for CR groups in TCGA-LGG and TCGA-GBM using Kaplan-Meier Curves (Log-Rank test, P <0.0001 in LGG; P <0.0001 in GBM). **(B)** Survival analyses for CR groups in LGG and GBM from CGGA cohort using Kaplan-Meier Curves (Log-Rank test, P <0.0001 in LGG; P =0.0068 in GBM).
