# Supplementary figures and images for: Circadian Regulation Patterns With Distinct Immune Landscapes in Gliomas Aid in the Development of a Risk Model to Predict Prognosis and Therapeutic Response
Source: Front Immunol. 2022 Jan 6;12:797450. doi: 10.3389/fimmu.2021.797450 (PMC8770819; doi:10.3389/fimmu.2021.797450)

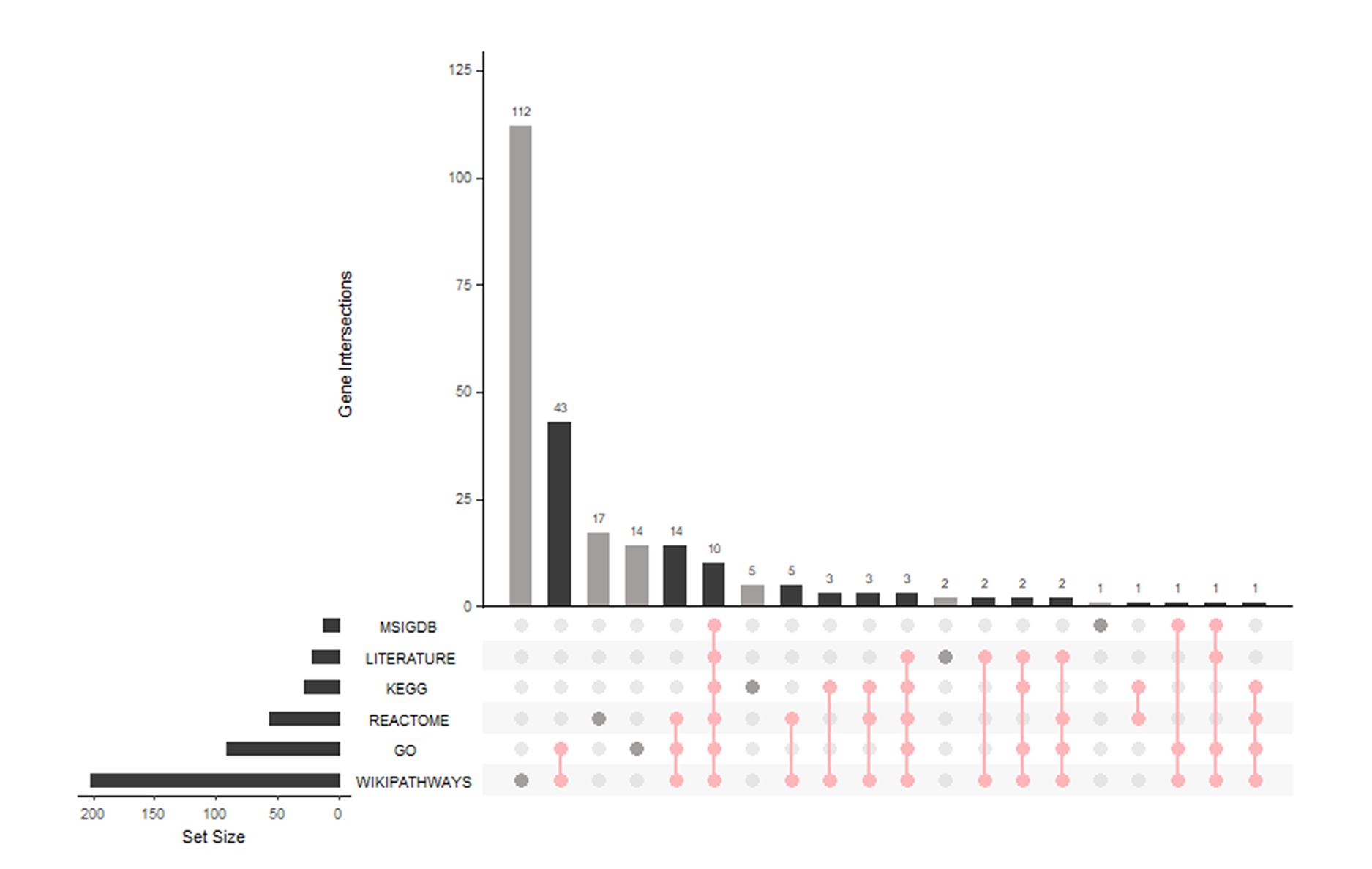

Supplement: Supplementary Figure 1 — Six CR gene sets were concluded from five datasets and review literatures. The gene annotated in not less than two circadian gene sets was defined as a CR gene. A total of 91 acknowledged CR genes were concluded here. The number of pick circles connected by lines indicated the number of times a CR gene appears in different gene sets; The number of genes sharing the same annotation pattern was marked at the top of the corresponding bar chart. The bar chart on the left showed the number of circadian genes in each gene set. [file Image_1.tif]

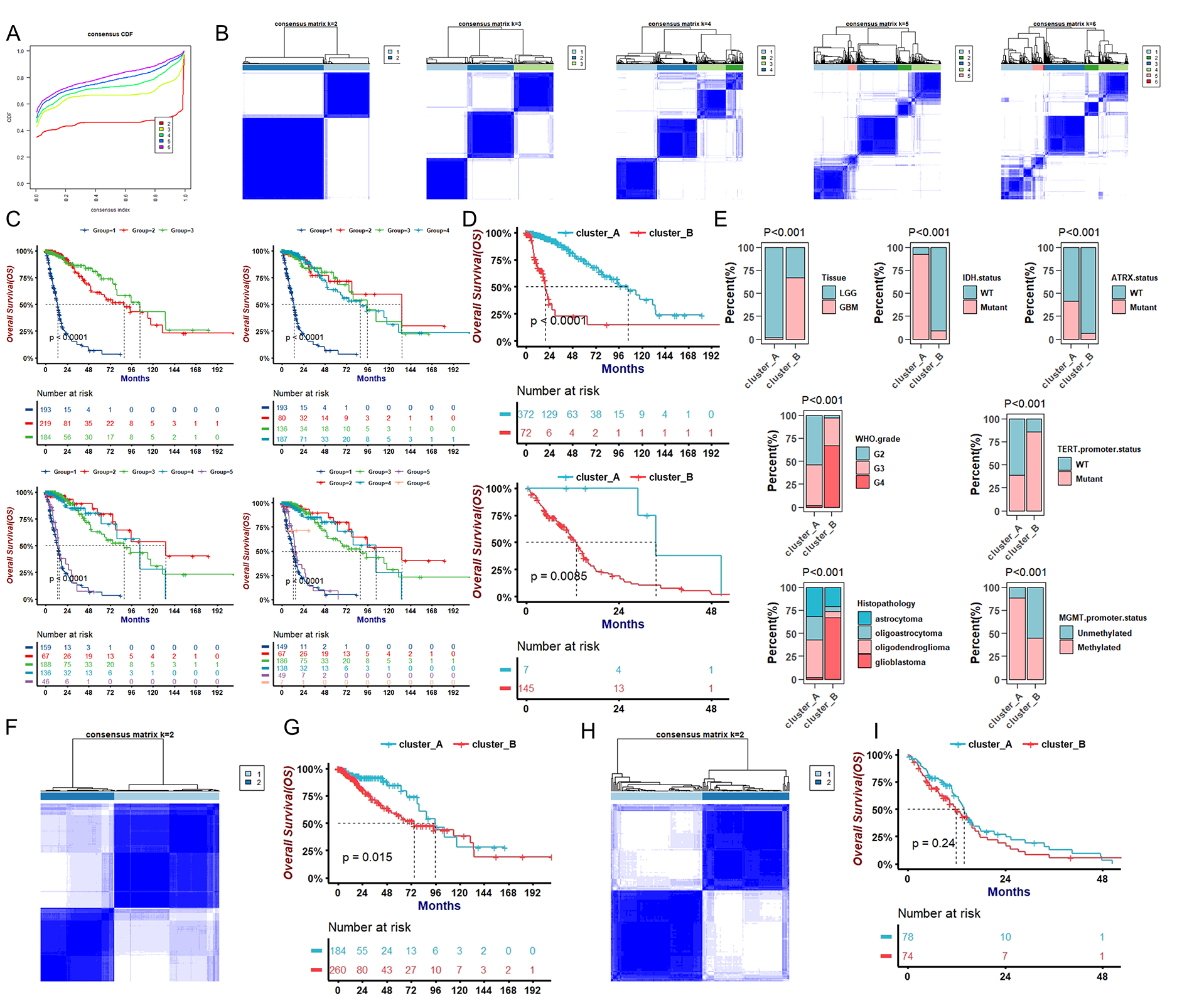

Supplement: Supplementary Figure 2 — Identification of two circadian patterns mediated by CR genes in TCGA cohort. (A) CDF plot showing a real random variable of its probability distribution based on consensus scores for each k (from 2 to 6, indicated by different colors) in TCGA cohort. (B) Consensus matrices of TCGA cohort for each k (k = 2–6), displaying the clustering stability using 1000 iterations of hierarchical clustering. (C) Survival analyses for CR patterns in TCGA cohort using Kaplan–Meier curves for OS when k = 3–6 (Log-Rank test: P < 0.0001). (D) Kaplan–Meier curves for OS of TCGA-LGG cohort (upper) including 372 cases in CR cluster-A and 72 cases in CR cluster-B (Log-Rank test: P < 0.0001), and TCGA-GBM (lower) including 7 cases in CR cluster-A and 145 cases in CR cluster-B (Log-Rank test: P = 0.0085). (E) The proportion of representative clinical parameters of patients in CR cluster A and B relevant to Figure 2A (Fisher’ exact test: P <0.001). (F) Consensus matrices of TCGA-LGG cohort for k = 2, displaying the clustering stability using 1000 iterations of hierarchical clustering. (G) Kaplan–Meier curves for OS of patients in TCGA-LGG cohort when k = 2. (H) Consensus matrices of TCGA-GBM cohort for k = 2, displaying the clustering stability using 1000 iterations of hierarchical clustering. (I) Kaplan–Meier curves for OS of patients in TCGA-GBM cohort when k = 2. [file Image_2.tif]

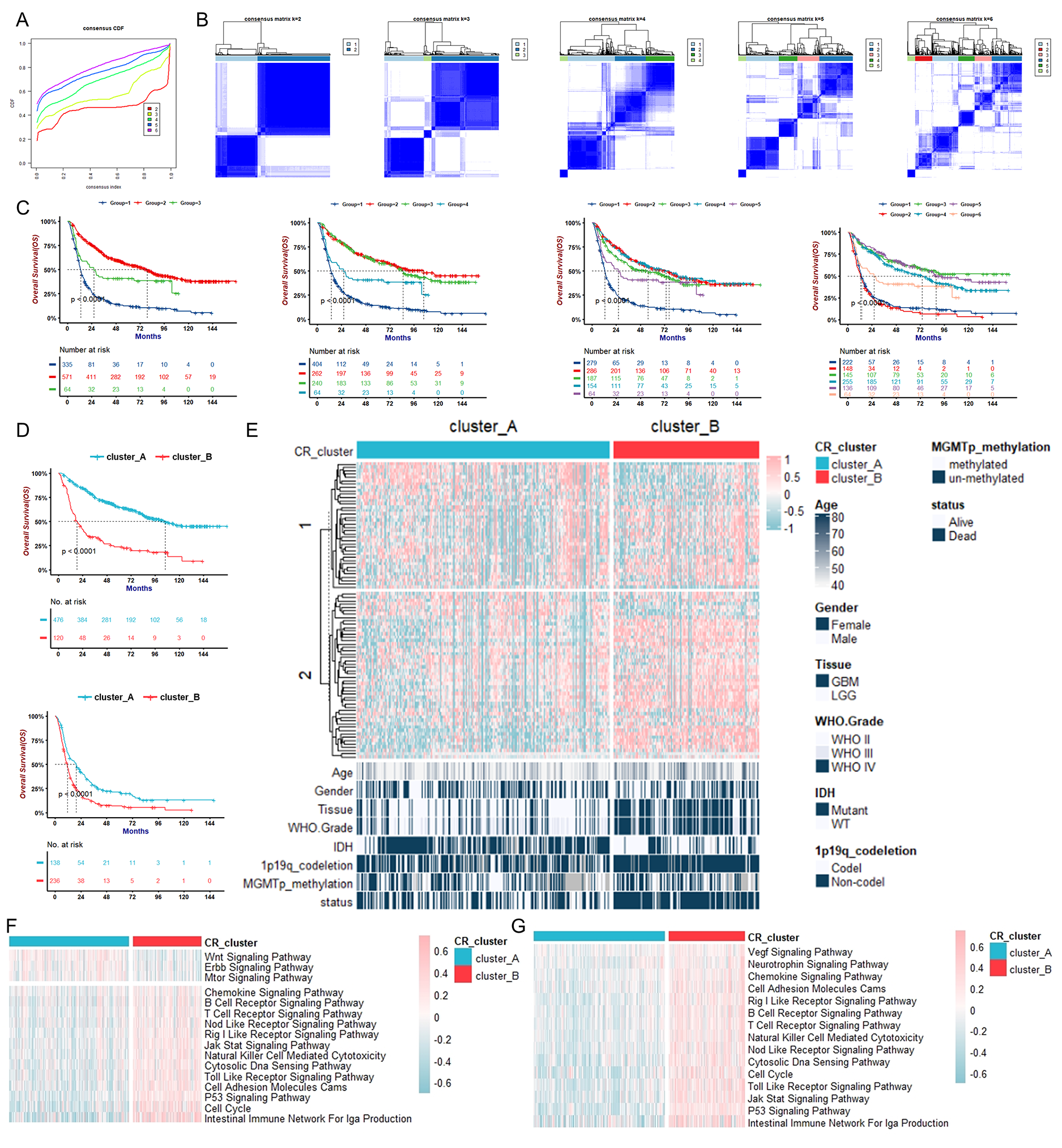

Supplement: Supplementary Figure 3 — Verification of two circadian patterns in CGGA cohort and functional annotations of CR clusters. (A) CDF plot showing a real random variable of its probability distribution based on consensus scores for each k (from 2 to 6, indicated by different colors) in CGGA cohort. (B) Consensus matrices of CGGA cohort for each k (k = 2–6). (C) Kaplan–Meier curves for OS of CGGA cohort when k = 3–6 (Log-Rank test: P < 0.0001). (D) Kaplan–Meier curves for OS of LGG patients in CGGA cohort (upper) including 476 cases in CR cluster-A and 120 cases in CR cluster-B (Log-Rank test: P < 0.0001), and GBM patients in CGGA cohort (lower) including 138 cases in CR cluster-A and 236 cases in CR cluster-B (Log-Rank test: P < 0.0001). (E) Unsupervised clustering of 91 CR genes for 970 glioma patients in CGGA cohort resulted in two CR clusters. Age, gender, tissue, WHO grade, IDH status, 1p/19q codeletion status, MGMT promoter status, and survival status are shown as patient annotations. (F, G) GSVA enrichment analyses in TCGA (F) and CGGA (G) cohorts showing the activation states of KEGG pathways in two CR clusters. Activated pathways, pink; Inhibited pathways, blue. [file Image_3.tif]

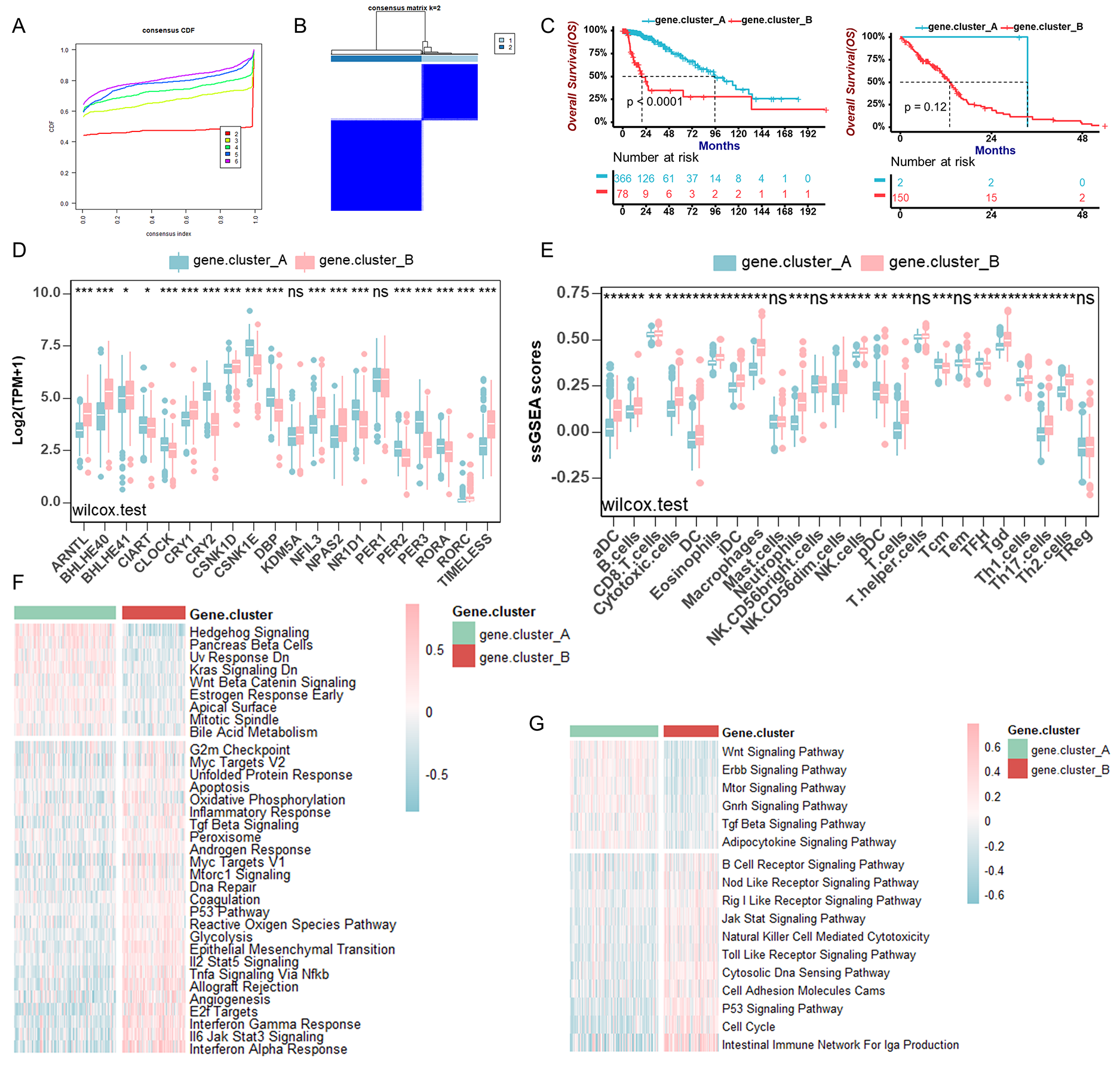

Supplement: Supplementary Figure 4 — Generation of circadian gene clusters and functional annotations of CR gene clusters. (A) Consensus matrices of TCGA cohort for k = 2. (B) CDF plot showing a real random variable of its probability distribution based on consensus scores for each k (from 2 to 6, indicated by different colors) in TCGA cohort. (C) Kaplan–Meier curves for OS of TCGA-LGG patients (left) and TCGA-GBM patients (right), respectively. (D) The mRNA expression level of core CR genes in gene cluster A and B. Gene cluster A, blue; Gene cluster B, pink. The asterisks represented the statistical p value (Wilcoxon test: ns, P >0.05; *, P <0.05; ***, P <0.001). (E) The ssGSEA score of TME cells in two CR gene clusters of TCGA cohort. (Wilcoxon test: ns, P>0.05; **, P <0.01; ***, P <0.001). (F, G) GSVA enrichment analyses in TCGA cohort showing the activation states of Hallmark pathways (MSigDB) (F) and KEGG pathways (G) in two CR gene clusters. Activated pathways, pink; Inhibited pathways, blue. [file Image_4.tif]

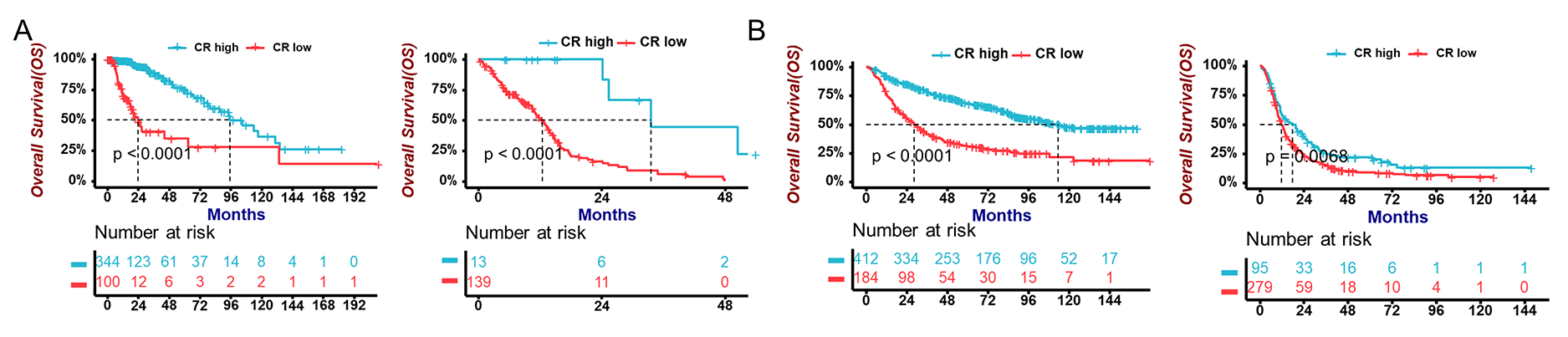

Supplement: Supplementary Figure 5 — Survival analyses for CR groups. (A) Survival analyses for CR groups in TCGA-LGG and TCGA-GBM using Kaplan-Meier Curves (Log-Rank test, P <0.0001 in LGG; P <0.0001 in GBM). (B) Survival analyses for CR groups in LGG and GBM from CGGA cohort using Kaplan-Meier Curves (Log-Rank test, P <0.0001 in LGG; P =0.0068 in GBM). [file Image_5.tif]
